# Case report: Three characteristics of tyrosine kinase inhibitor-associated cerebrovascular stenosis. High threshold for infarction, atypical infarct area, and vascular recoverability under the use of ponatinib

**DOI:** 10.3389/fstro.2024.1367869

**Published:** 2024-04-03

**Authors:** Akira Hanazono, Masamichi Abe, Shuntaro Togashi, Teruko Takahashi, Naoto Takahashi, Masashiro Sugawara

**Affiliations:** ^1^Department of Neurology, Akita University Graduate School of Medicine, Akita, Japan; ^2^Department of Neurosurgery, Akita University Graduate School of Medicine, Akita, Japan; ^3^Department of Hematology, Nephrology, and Rheumatology, Akita University Graduate School of Medicine, Akita, Japan

**Keywords:** chronic myeloid leukemia, moyamoya disease, functional neurological disorder, Sonoo abductor test, arm drop test, bosutinib, cortical infarction

## Abstract

While tyrosine kinase inhibitors (TKI)-associated cerebral vascular stenosis (CVS) exhibit distinct mechanisms compared to conventional stroke in basic research, the clinical strategy remains nearly the same other than TKI-switching. We present the case of a 22-year-old female with chronic myeloid leukemia without stroke risk factors, who developed ponatinib-associated CVS. Three potential characteristics of TKI-associated CVS were identified: a heightened threshold for infarction, an atypical infarct area, and vascular recoverability. Specifically, brain computed tomography remained normal despite 20 h of severe hemiplegia. The ischemic distribution was confined in gray matter and the anterior cerebral artery territory on magnetic resonance imaging, despite severe stenosis of the internal carotid artery. Ischemic changes resolved within 10 days and arterial stenosis improved after ponatinib withdrawal. These unique features, distinct from typical stroke, could lead to misdiagnosis as non-organic neurological disorders or other conditions in ponatinib-treated patients.

## 1 Introduction

Tyrosine kinase inhibitors (TKIs) improved the prognosis of chronic myeloid leukemia (CML). However, some of these anti-angiogenic drugs are associated with cerebrovascular stenosis (CVS) and cardiovascular adverse events (Moslehi and Deininger, 2015; Manouchehri et al., 2020) especially in ponatinib (Haguet et al., 2020). Nevertheless, some treatment-resistant patients with CML having specific mutations such as T315I, must rely on this angiopathic TKI (Moslehi and Deininger, 2015; Haguet et al., 2020; Manouchehri et al., 2020). On the other hand, many basic researches suggested that CVS associated with TKIs may be attributed to the direct effect on endothelium cells, unlike conventional atheromatous causes of stroke (Haguet et al., 2020; Madonna et al., 2020). However, the clinical strategy remains almost the same as conventional stroke other than TKI switching, even in the absence of reliable reasons (Moslehi and Deininger, 2015; Manouchehri et al., 2020). Therefore, clarifying the clinical difference between TKI-associated CVS and conventional stroke is important.

We present a case of a 22-year-old patient with CML without antecedent risks for stroke, who developed TKI (ponatinib)-associated CVS. This case report highlighted a distinct aspect of ponatinib-associated CVS, which was a high threshold for infarction, an atypical infarct area, and vascular recoverability after TKI withdrawal. In addition, due to these differences from typical strokes, this report also cautioned the misdiagnosis of TKI-associated brain ischemia as non-organic palsy or other diseases.

## 2 Case presentation

**Figure 1:**
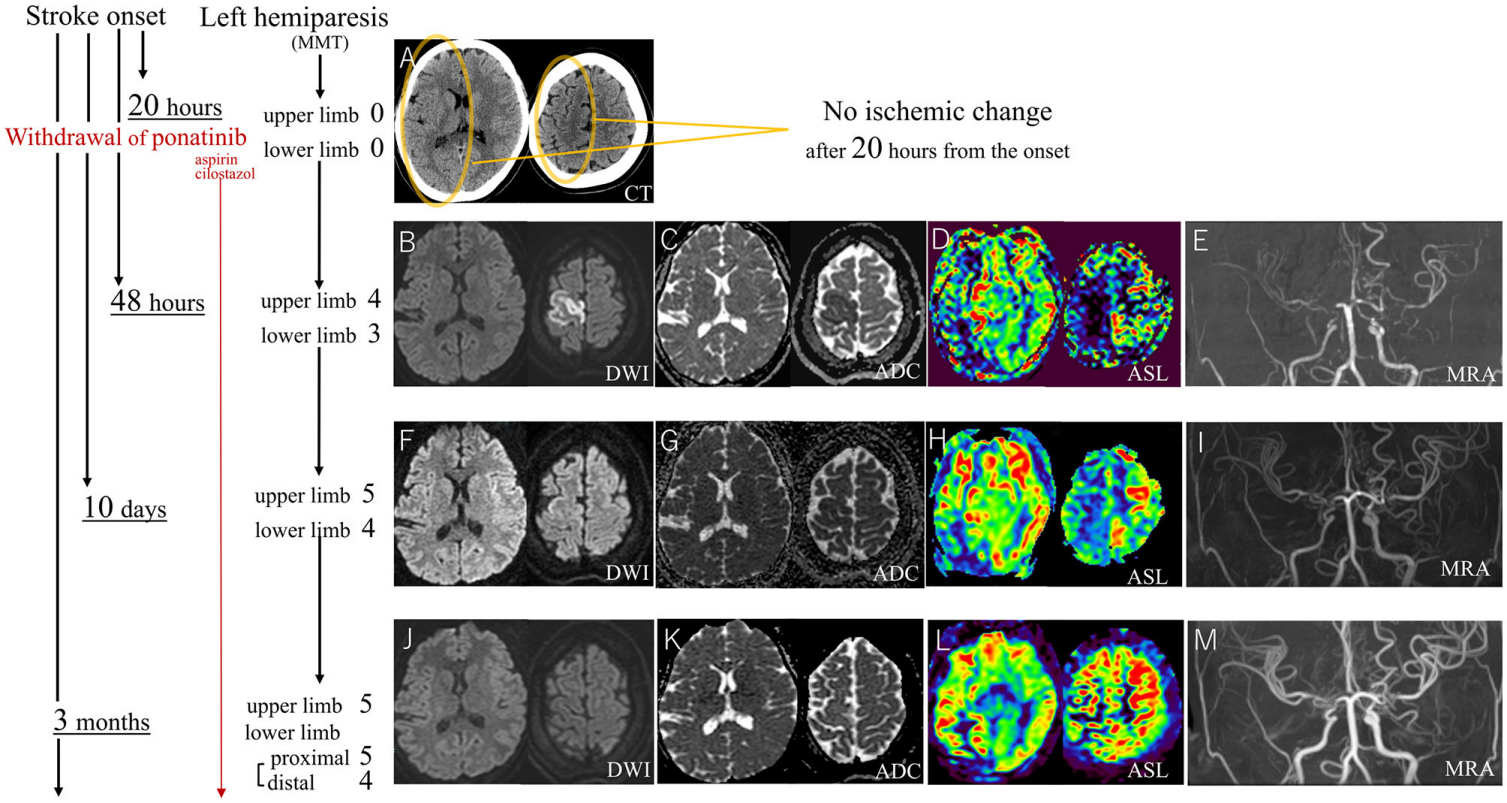
After 20 h of stroke onset with severe MMT-0 left hemiplegia, the CT scan shows no ischemic changes **(A)**. DWI and ADC on MRI at 48 h from the onset show gray matter-confined infarction at the right ACA area **(B, C)** which is an atypical lesion in ICA stenosis **(E)**. Ten days after the stroke onset, the MRI shows the almost disappeared ischemic lesions **(F, G)**, and after 3 months, these lesions completely improved **(J, K)**, ASL and MRA show striking improvement after the withdrawal of ponatinib **(D, E, H, I, L, M)**. MMT, manual muscle test; CT, computed tomography; DWI, diffusion weighted imaging; ADC, apparent diffusion coefficient; MRI, magnetic resonance imaging; ACA, anterior cerebral artery; ICA, internal carotid artery; ASL, arterial spin labeling; MRA, magnetic resonance angiography.

The case was a 22-year-old female patient, who has a history of ~3 years of CML, managed with bosutinib for around 6 months, followed by about a 2-year course of ponatinib because of resistance to previous TKI. Then, she presented with recurring numbness without sudden palsy in both of her upper limbs, predominantly on the left side, for about 8 months. Because this symptom was similar to cervical spinal stenosis, it was treated symptomatically. Notably, she lacked atherosclerotic risk factors such as hyperlipidemia, hypertension, diabetes, smoking, or cardiac conditions like atrial fibrillation even after the use of ponatinib, and there was no evident family history of stroke. However, her transient sensory symptoms repeated and increased, and one day, she experienced left hemiparesis, resulting in a bedridden status. At arrival at our hospital, the patient displayed hypertension with an elevated heart rate (blood pressure: 165/91 mmHg, heart rate: 106/min), but other vital signs were within the normal range. Her hemiplegia was so severe that Manual Muscle Testing (MMT) was almost 0 (no muscle contractions), but the Babinski sign was negative. Despite the severity and duration (about 20 h) of her symptoms, a brain CT scan did not exhibit any ischemic changes including early CT signs as confirmed by emergency physicians, radiologists, and neurologists (Figure 1A). Given the absence of abnormalities on the CT scan, an emergent Magnetic Resonance Imaging (MRI) was postponed to the next day because of the overbooking in our hospital. Besides, her paralyzed left arm was able to resist falling to her body during the “arm drop test,” and her completely paralyzed left leg was moved paradoxically by the “Sonoo abductor test.” Thus, functional (psychological) hemiparesis was considered. She was admitted to our hospital, and as a precaution, ponatinib was discontinued. The following day, her hemiparesis showed improvement (MMT score 4 in the upper limb and 3 in the lower limb), but, the first brain MRI showed abnormalities such as high-intensity diffusion-weighted imaging (DWI) and low apparent diffusion coefficient (ADC) in the cortex of the right anterior cerebral artery (ACA) area, severe bilateral stenosis of the distal internal carotid artery (ICA), and severe perfusion decrease in the right hemisphere on arterial spin labeling (ASL; Figures 1B–E). Digital subtraction angiography (DSA) showed not only these arterial stenoses but collateral flows with transdural, choroidal, and leptomeningeal anastomoses. These appeared to be moyamoya disease but did not meet the diagnostic criteria due to the lack of basal vascular networks (Kuroda et al., 2022). Given these abnormalities, cilostazol (200 mg per day) and aspirin (100 mg per day) were initiated. Laboratory tests did not indicate the causes of arterial stenosis with anastomoses such as hyperthyroidism, vasculitis, collagen disorders, infections, or coagulation abnormalities. Then, MRI showed a notable improvement in the vanished high DWI/low ADC lesions within 10 days (Figures 1F, G), and after 3 months, these lesions completely improved (Figures 1J, K). Follow-up DSA further displayed improved cerebral flow, particularly in the right ACA. As time advances, decreased flow on the ASL and bilateral distal ICA stenosis on MRA were both ameliorated (Figures 1D, E, H, I, L, M). Considering the rapid improvements in cerebral blood flow and arterial stenoses, it could not be solely attributed to the effects of cilostazol and aspirin which was continued throughout the clinical course. Furthermore, as this remission completely ruled out moyamoya disease, ponatinib, and its withdrawal may be the primary causes of stroke and improvement. With the rehabilitation, her hemiplegia notably improved to the MMT score of 5, except for mild weakness around her left ankle joint. Despite the residual perfusion decrease in the area of the right posterior cerebral artery on ASL and MRA (Figures 1L, M), no ischemic changes were detected in this area on fluid-attenuated inversion recovery (FLAIR) throughout the clinical course. Then, ponatinib was replaced with asciminib following its recent approval in Japan, and vascular stenoses did not recur.

## 3 Discussion

This case highlights three unique observations of ponatinib-associated CVS: high threshold for infarction, atypical infarct area, and vascular recoverability. Firstly, typically, ischemic symptoms manifest ischemic CT signs within a few hours, and prolonged time enhances the likelihood of detecting ischemic changes (Heiss, 2011; Gao et al., 2017; van Poppel et al., 2022). However, in contrast to conventional strokes, the CT scan in this case showed no abnormalities even after 20 h, despite the patient experiencing severe MMT-0 bedridden hemiplegia. This atypical manifestation caused misdiagnosis. Furthermore, DWI hyperintense lesions were selected only in the cortical lesion without white matter, suggesting the perfusion decrease remained moderate because white matter is known to be more durable to ischemia (Chen et al., 2019). Consistently, this incomplete ischemia on MRI nearly disappeared within 10 days. Additionally, antecedent transient ischemic attacks on bilateral upper limbs without residual symptoms over an extended 8 months, also suggested a high threshold for the infarction, and it was confusing with chronic cervical spinal stenosis. Similarly, the absence of residual ischemic change on FLAIR in the hypoperfusion area indicated the durability against ischemia. Corresponding to our case, some cases of TKI-associated CVS also showed no infarctions without any symptoms despite the severe arterial stenoses (Hirayama et al., 2022). If this observation—fewer radiological and clinical manifestations in ponatinib-associated CVS due to the resistance to infarction—holds true universally, it challenges the drug information and reviews recommending CVS evaluation only in symptomatic patients (Moslehi and Deininger, 2015; Manouchehri et al., 2020), because our observations suggested this approach may be too passive. Therefore, cranial angiography (preferably a less invasive MRA if possible) might become a necessary examination in the future before initiating and after prescribing TKIs, even without neurological symptoms. The second observation is the atypical infarct area of isolated ACA-ischemia on DWI because ICA stenosis like our case contributed to only 6% of ACA area infarction in conventional strokes (Kang and Kim, 2008). Atypical ACA-area involvement due to ipsilateral proximal middle cerebral artery stenosis by TKI (nilotinib) was also reported (unfortunately the authors did not mention atypical ACA-area involvement; Kakadia et al., 2021). In addition, compared to conventional stroke, the aforementioned selectivity of infarction in cortical lesions without white matter lesions was also atypical, and a similar trend might be also shown in another case with ponatinib (Spina et al., 2020). Thirdly, vascular recoverability after ponatinib-withdrawal. This was also noted in other ponatinib-associated CVS (Hirayama et al., 2022). Even though this might be affected by the youth of our case to some extent, typical atherosclerosis rarely improves spontaneously to such an extent. In addition, the initial developments of anastomoses like moyamoya disease suggested the stenoses lasted for a significant duration, and this excluded the vasospasm. If this recoverability is widely applicable, it cautioned against recent reports recommending invasive bypass surgery or stenting for TKI-associated CVS under the assumption of irreversibility (Suzuki et al., 2019; Hirayama et al., 2022; Rai and Hara, 2023). Because of the lack of human histopathological evaluations for TKI-associated CVS, the reasons for these three differences from typical stroke remain unclear. In addition, it's important to note that ponatinib is the most multitargeted inhibitor among several TKIs (Moslehi and Deininger, 2015; Manouchehri et al., 2020) and these three observations might be confined to ponatinib. Nonetheless, given this case was a young patient with no traditional stroke risk factors and prior bosutinib was deemed an unlikely cause of CVS (Moslehi and Deininger, 2015; Haguet et al., 2020), we believe these characteristics represent a pure manifestation of ponatinib with no other confounding factors. We propose two possible explanations for our observations.

The first explanation involves potential commonalities with moyamoya disease because the present case exhibited bilateral ICA stenoses with collateral flow. Sharing similarities with TKI's anti-angiogenic mechanisms, a recent report on moyamoya disease proposed that abnormal angiogenesis might be the fundamental etiology (Zhuo et al., 2019). In addition, radiological observations were also similar, as stenotic characteristics of moyamoya disease with donuts-like concentric pattern (Kim et al., 2013; Ya et al., 2020) were also reported in TKI-associated CVS (Hirschbuehl et al., 2019; Suzuki et al., 2019) and large vessel stenosis of the leg (Hirschbuehl et al., 2019), which differentiate from the typical eccentric atherosclerosis. Furthermore, clinical manifestations of moyamoya disease may also resemble the present TKI-associated case, featuring an extended asymptomatic period (Kuroda et al., 2023) possibly due to resistance to infarctions, atypical infarct areas irrespective of anatomical perfusion areas, and confined infarctions in gray matter (known as “gyral pattern,” especially in younger patients; Cho et al., 2011; Kuroda et al., 2023). Added to these similarities, some TKI-associated CVS was reported to be affected by the genetical abnormality (ring finger protein 213) of moyamoya disease (Uemura et al., 2020). Although only two reports suggested the connection to moyamoya disease in the long history of TKI (Zhuo et al., 2019; Uemura et al., 2020), future studies should focus on the etiological relationship between moyamoya disease and TKI-associated CVS. Additionally, since East Asia, including Japan, has a high prevalence of moyamoya disease, and the genetic abnormalities associated with moyamoya disease contribute to large-artery stenosis (Okazaki et al., 2019), Asian countries should exercise greater vigilance regarding cranial large arterial stenosis when using TKIs.

The second explanation, especially in tissue survivability under prolonged ischemia, may be linked to the anti-angiogenic effects of TKIs on atherosclerosis. Considering that angiogenesis is a primary contributor to plaque rupture (Sedding et al., 2018) and several tyrosine kinases mainly mediate the angiogenesis (Camaré et al., 2017), it may contribute to sudden onset and symptomatic completion. Thus, while TKIs induce slowly progressive vascular stenosis with unknown etiology, the acute onset of stroke might be prevented by anti-angiogenesis (like being left in limbo). In this context, we assume the developed collateral flow, even at the onset in the present case, might be the result of persistent but unfinished ischemia by TKI's anti-angiogenic effect.

On the other hand, it is crucial to acknowledge a clinical discrepancy that led to misdiagnosis. Despite the patient's MMT-0 left hemiparesis, these limbs were paradoxically movable during the arm drop test and Sonoo abductor test, which are commonly employed in evaluating functional/psychogenic movement disorders (Popkirov et al., 2020). It is worth postulating that these prolonged penumbra tissues were more reactive to these maneuvers than the voluntary movement at MMT, given that the penumbra is only a recoverable electrophysiological cell-function disturbance (Heiss, 2011). This phenomenon might be consistent with observations in rehabilitation divisions that utilize this kind of reflexive movement as a stimulator for recoverable ischemic brain tissues that could not react voluntarily (Pandian et al., 2012). Therefore, these paradoxical movements also suggested cerebral blood flow reduction was moderate enough for brain tissues to react to specific maneuvers like Sonoo abductor test, and to recover in the future.

## 4 Conclusion

To prevent misdiagnoses and inappropriate treatments, it is essential to recognize three characteristics associated with ponatinib-induced CVS: a high threshold for infarction, atypical infarct areas, and vascular recoverability. Additionally, the similarities observed in our case to moyamoya disease raise concerns about possible regional or genetic susceptibility to TKIs especially in East Asian countries, and suggest a part of the etiology of vascular stenosis. Therefore, strategies for managing TKI-associated CVS should be distinguished from those for typical strokes, considering not only the differences in prior basic research but also the clinical differences observed in the present case.

## Data availability statement

The original contributions presented in the study are included in the article/supplementary material, further inquiries can be directed to the corresponding author.

## Ethics statement

Ethical approval was not required for the studies involving humans because this case was a case report. The studies were conducted in accordance with the local legislation and institutional requirements. The participants provided their written informed consent to participate in this study. Written informed consent was obtained from the individual(s) for the publication of any potentially identifiable images or data included in this article.

## Author contributions

AH: Writing – original draft, Writing – review & editing, Conceptualization, Project administration. MA: Data curation, Supervision, Visualization, Writing – review & editing. ST: Data curation, Supervision, Writing – review & editing. TT: Supervision, Writing – review & editing, Data curation. NT: Supervision, Writing – review & editing, Data curation. MS: Supervision, Writing – review & editing.
